# Supplementary material for: Allopatric divergence of cooperators confers cheating resistance and limits effects of a defector mutation
Source: BMC Ecol Evol. 2022 Dec 12;22:141. doi: 10.1186/s12862-022-02094-7 (PMC9746145; doi:10.1186/s12862-022-02094-7)
Supplement: Supplementary file 1 — Additional file 1: Supplemental Information. [file 12862_2022_2094_MOESM1_ESM.pdf]

Supplemental Information for:

Allopatric divergence of cooperators confers cheating resistance and limits effects of a defector mutation

Kaitlin A. Schaal*[1], Yuen-Tsu Nicco Yu[1], Marie Vasse[1,2], and Gregory J. Velicer[1]

[1]Institute of Integrative Biology, ETH Zürich
[2]Institute MIVEGEC (UMR 5290 CNRS, IRD, UM), 34394, Montpellier, France

* Corresponding author. Email: contact@kaitlinschaal.com

Fig. S1. Absolute sporulation levels of DK5208, GJV1 and 23 natural isolates in pure culture, DK5208 in mixture with all other strains and total spore production of all mixes

Fig. S2. Absolute sporulation levels of DK5208, GJV1, and all MyxoEE-3 lab-evolved clones in pure culture, DK5208 in mixture with all other strains, and total spore production of all mixes

Fig. S3. *csgA* sequences of *M. xanthus* strains DK1622/GJV1 and four natural isolates disrupted at *csgA* in this study

Fig. S4. Absolute sporulation levels of i) DK5208, GJV1, and four natural isolates in pure culture, ii) each of five new c*sgA* mutants in 1:99 mixture with all five parental strains, and 3) total spore production of all mixes

Fig. S5. One-way mixing effects on *csgA* mutants

Fig. S6. Social defects, but no cheating, of different *csgA* mutants in the GJV1 background

Table S1. *M. xanthus* strains

Table S2. History and mutations of MyxoEE-3 lab-evolved strains

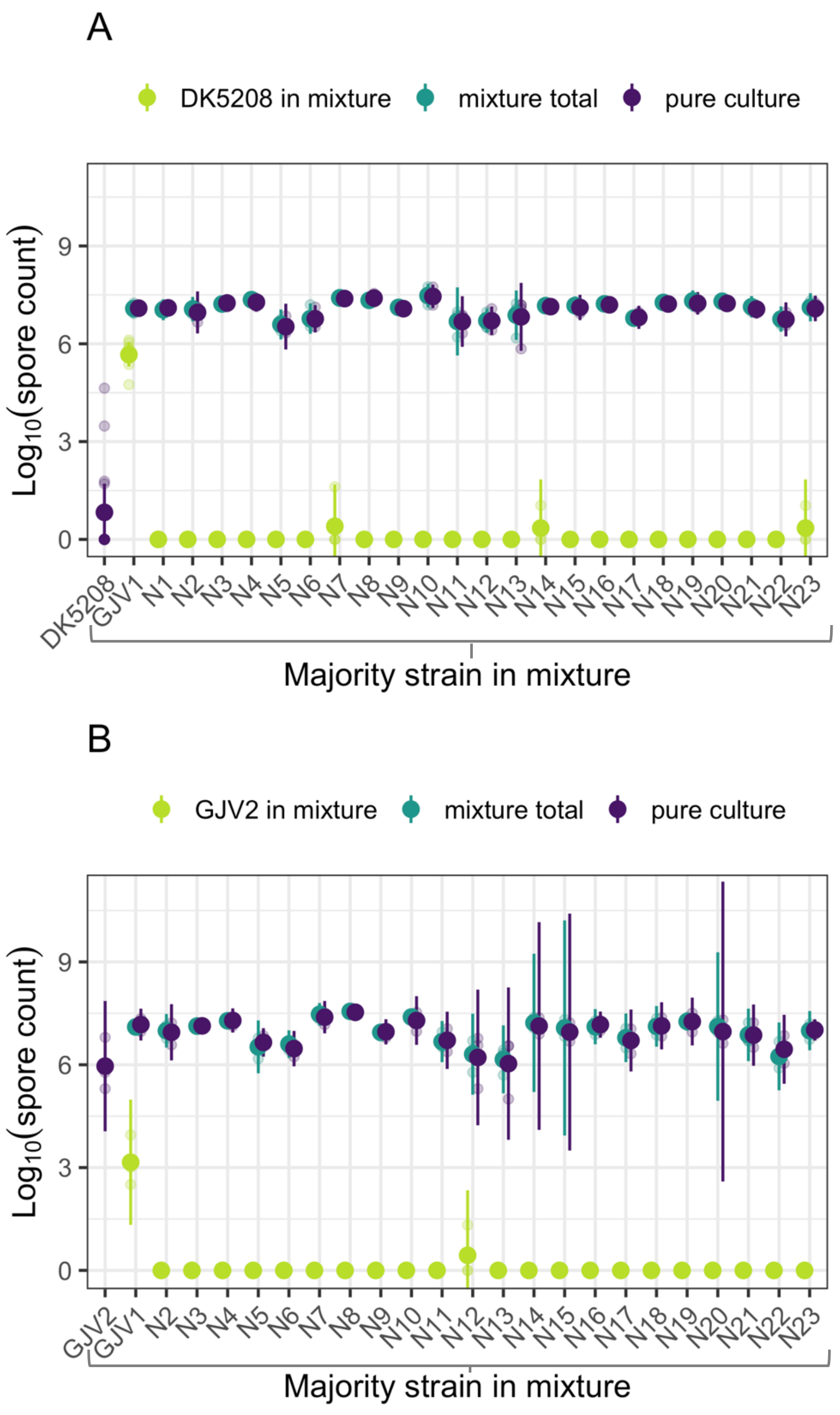

Fig. S1. Absolute sporulation levels of DK5208, GJV1 and 23 natural isolates in pure culture, DK5208 in mixture with all other strains and total spore production of all mixes. (A) DK5208 in mixture with GJV1 and 23 natural isolates. (B) GJV2 in mixture with GJV1 and 22 natural isolates. DK5208 and GJV2 started as 1% in all mixed groups. Small circles represent individual replicate estimates, large circles show cross-replicate averages, and error bars show 95% confidence intervals; 3-4 biological replicates for natural isolate mixes, 8 for GJV1.

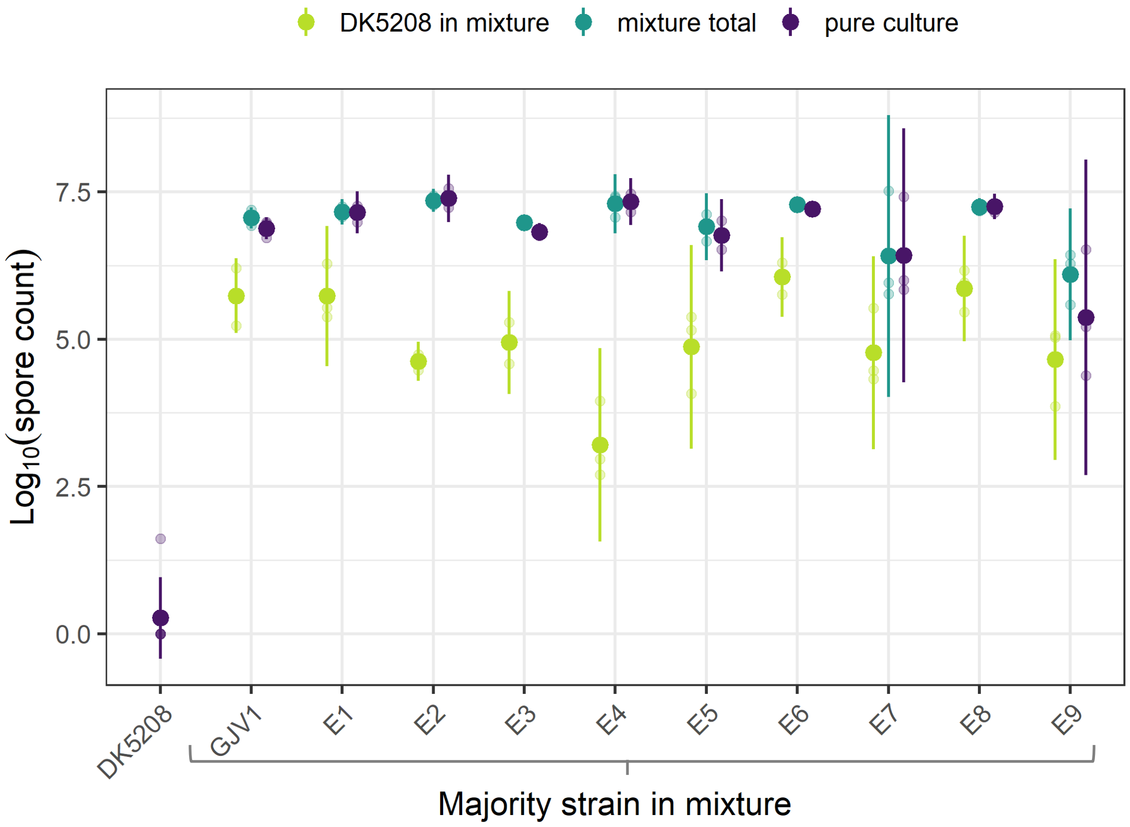

Fig. S2. Absolute sporulation levels of DK5208, GJV1, and all MyxoEE-3 lab-evolved clones in pure culture, DK5208 in mixture with all other strains, and total spore production of all mixes. DK5208 started as 1% in all mixed groups. Small circles represent individual replicate estimates, large circles show cross-replicate averages, and error bars show 95% confidence intervals; 3-4 biological replicates.

A

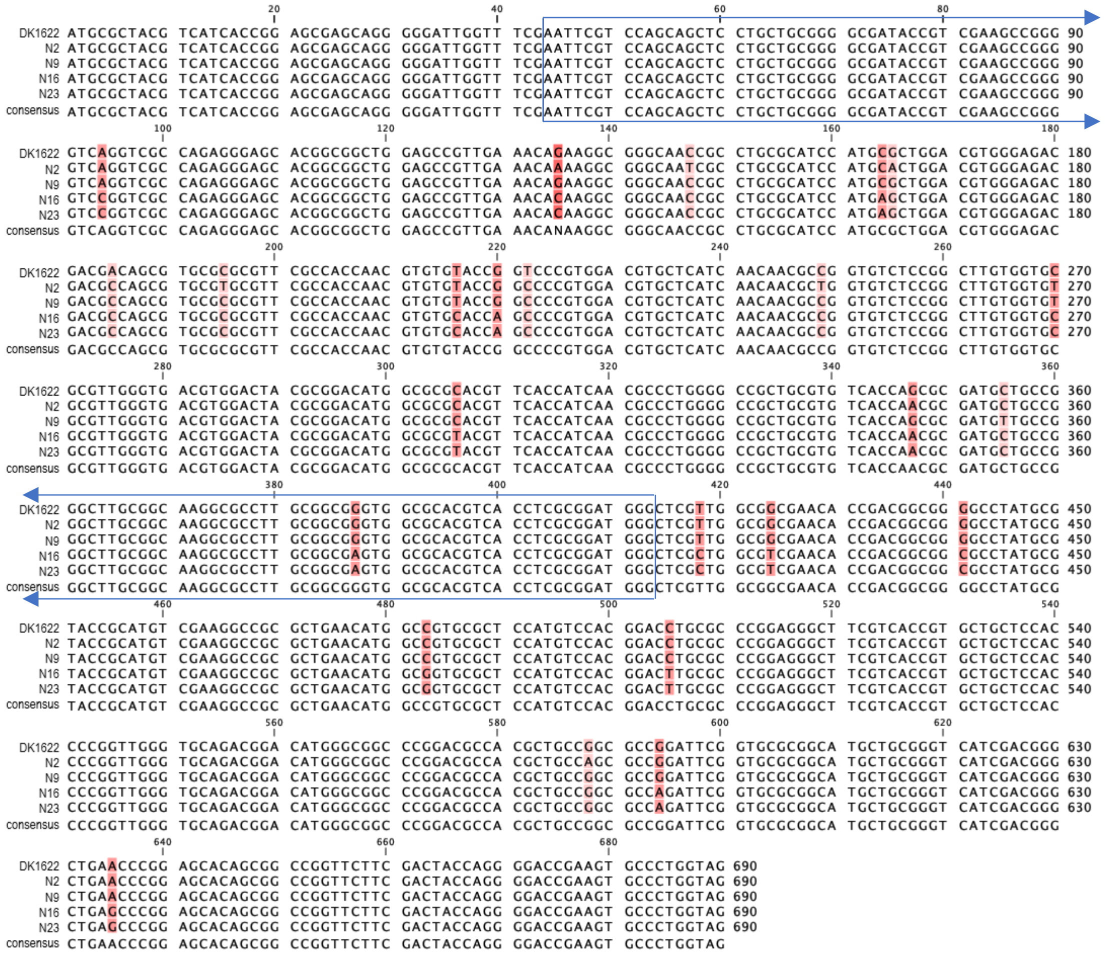

B

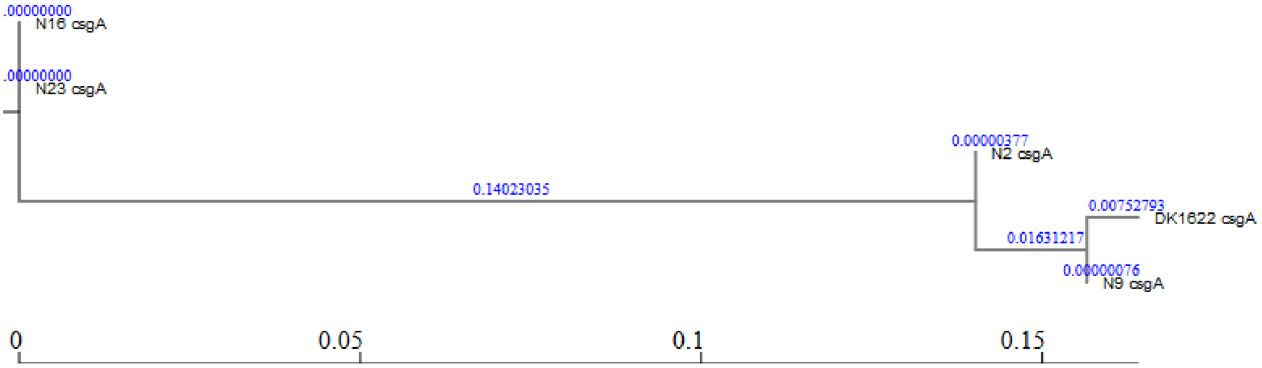

Fig. S3. *csgA* sequences of *M. xanthus* strains DK1622/GJV1 and four natural isolates disrupted at *csgA* in this study. (A) Sequence alignment, where red shading indicates polymorphic sites, with darkness reflecting the degree of polymorphism. Boxed regions indicate the beginning and end of the region used as inserts in plasmid construction. (B) Maximum likelihood tree of *csgA* sequences, where blue numbers indicate branch lengths in substitutions per site. The scale bar is in substitutions per site.

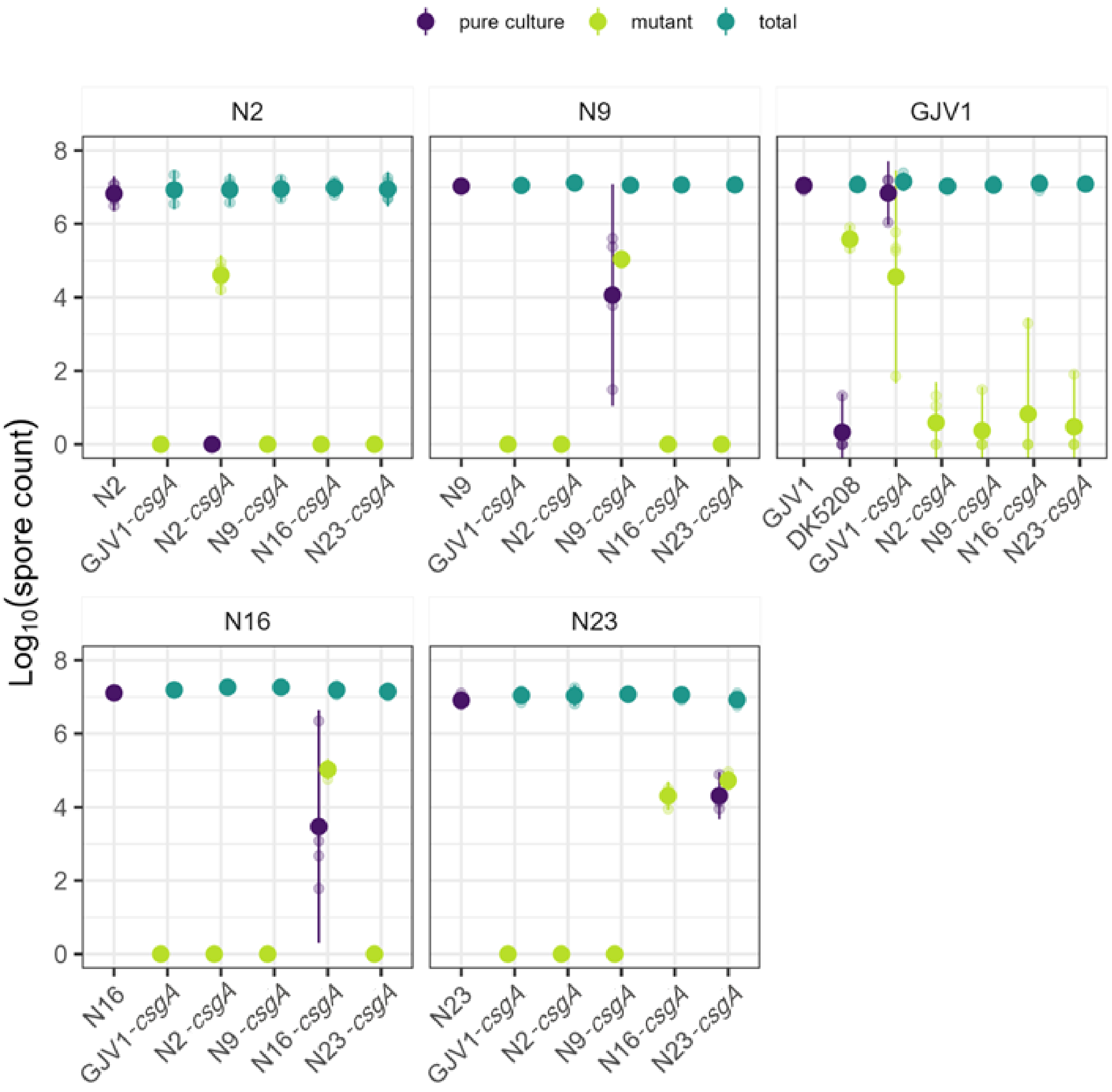

Fig. S4. Absolute sporulation levels of i) DK5208, GJV1, and four natural isolates in pure culture, ii) each of five new c*sgA* mutants in 1:99 mixture with all five parental strains, and 3) total spore production of all mixes. Small circles represent individual replicate estimates, large circles show cross-replicate averages, and error bars show 95% confidence intervals; 4 biological replicates.

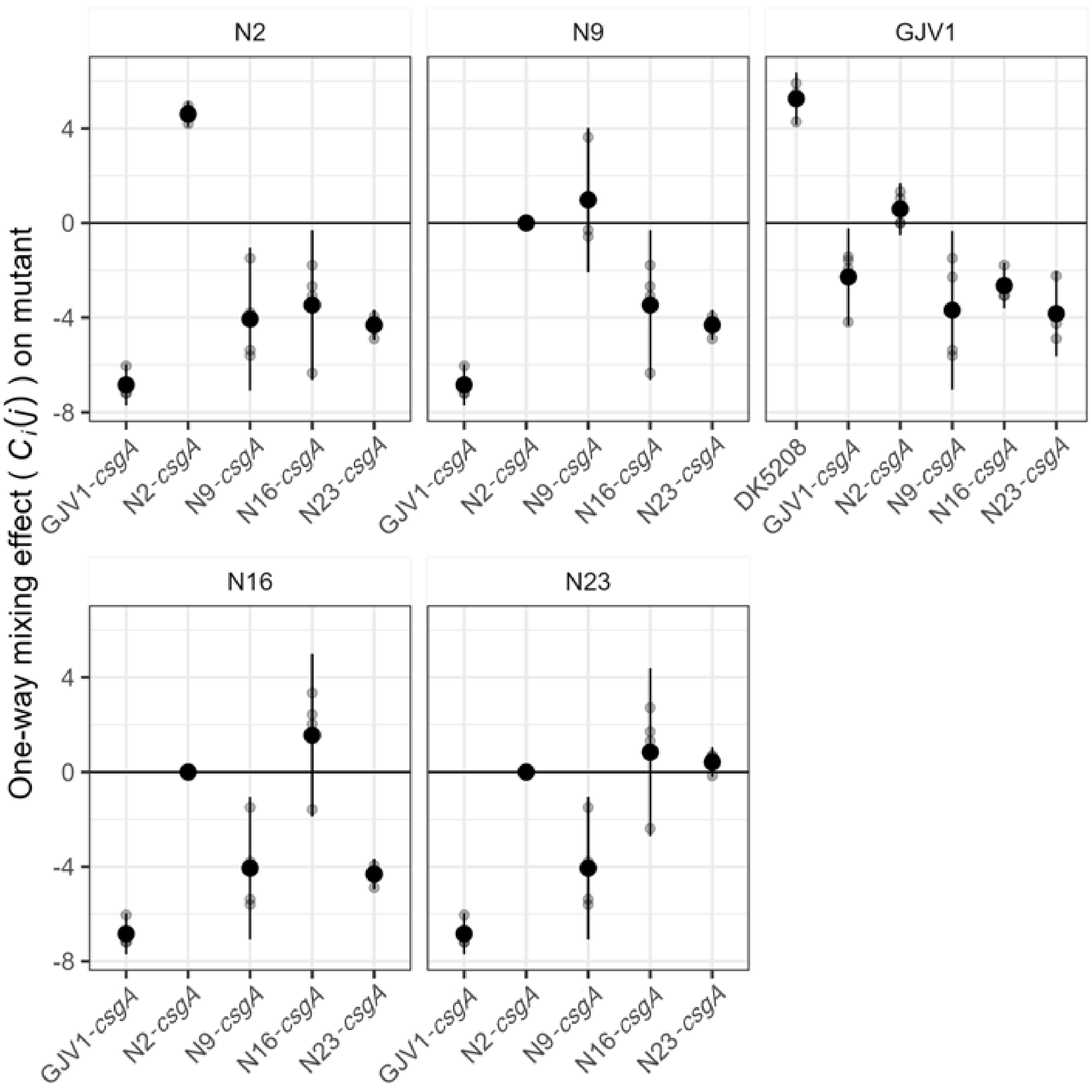

Fig. S5. One-way mixing effects on *csgA* mutants. We calculated the degree to which each *csgA* mutant sporulated better or worse in mixture with each proficient strain than it did in pure culture with the parameter $C_i(j)$ (see Methods). Small grey circles represent individual replicate estimates, large black circles show cross-replicate averages, and error bars show 95% confidence intervals; 4 biological replicates.

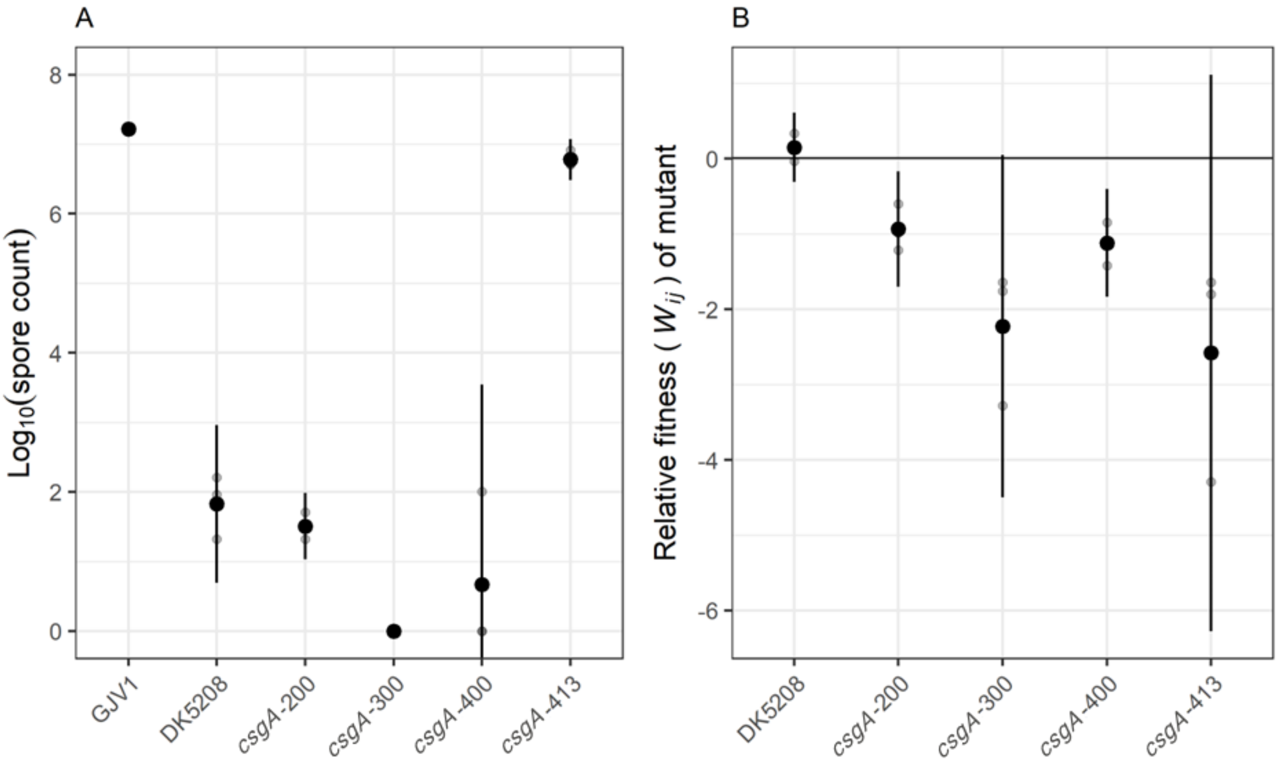

Fig. S6. Social defects, but no cheating, of different *csgA* mutants in the GJV1 background. We constructed three additional *csgA* mutants in a similar way to that reported in the Materials and Methods for construction of the *csgA*-413 mutants. We amplified a 200-bp, 300-bp, and 400-bp fragment of GJV1's native *csgA* allele using one forward primer (5'-TCATCACCGGAGCGAGCAGG-3'; genome positions 1520209-1520228) and three reverse primers, one each for the 200-bp fragment (5'-CACACGTTGGTGGCGAACGC-3'; genome positions 1520394-1520413), the 300-bp fragment (5'-GCCATGTCCGCGTAGTCCAC-3'; genome positions 1520481-1520500), and the 400-bp fragment (5'-CCATCCGCGAGGTGACGTGCGCCA-3'; genome positions 1520587-1520610). We continued with plasmid and mutant construction as reported in the Materials and Methods. We chose these three fragment lengths because we observed that our previous construct, GJV1-*csgA* (here referred to as *csgA*-413) had a different pure-culture sporulation phenotype from the existing defector strain DK5208, and we wanted to examine whether this was due to the location of the plasmid insertion in our mutant (413) compared to the transposon insertion in DK5208 (217). We chose fragment lengths that would interrupt *csgA* at positions covering the distance between the two existing mutants. (A) All of the mutants except for *csgA*-413 are developmentally defective compared to their parent strain GJV1 in mixture (p-values < 0.001, *csgA*-413 $p = 0.9$, post-hoc Tukey HSD test). Strain *csgA*-300 is more defective than any other mutant except *csgA*-400 (p-values < 0.035, *csgA*-400 $p = 0.62$, same post-hoc Tukey HSD test). Strain *csgA*-413 is the least defective of all the mutants (p-values < 0.001, same post-hoc Tukey HSD test). (B) All of the mutants constructed in this work show low relative fitness in mixture with GJV1. DK5208 is not statistically shown to cheat in this assay ($p = 0.15$, one-sided t-test for $W_{ij} > 0$). There is slight statistical evidence that the other mutants have reduced fitness relative to GJV1 during this interaction (mean W_{ij} values < 0, 0.05 < p-values < 0.1, 4 two-sided t-tests against 0 with Bonferroni-Holm correction). Mutants *csgA*-300 and *csgA*-413 have lower relative fitness than does DK5208 (p-values < 0.031, post-hoc Tukey HSD test). Given that they are the lowest- and highest-sporulating mutants, respectively, this suggests that the relationship among length of intact *csgA* gene, social defection phenotype (*i.e.*, amount of spores produced in pure culture), and fitness when mixed with parent (potential for cheating) is complex and nonlinear. Error bars are 95% confidence intervals. Black dots are averages of three biological replicates (grey dots).

Table S1. *M. xanthus* strains. Here we list the original published or new nomenclature of each strain and the simplified nomenclature used in this paper.

Original or formal name	Ref.	Referred to here as	Genetic manipulation	Resistance	Geographic origin
DK5208/LS523	[1, 2]	DK5208	*csgA*::Tn5-132	oxytetracycline	[a]
GJV1	[3, 4]	GJV1	-	-	[a]
GJV2	[3, 5, 6]	GJV2	spontaneous rif^R mutation	rifampicin	-
MyxoEE-3 P02 cycle 40 clone 1 [b]	[7–9]	E1	-	rifampicin	-
MyxoEE-3 P03 cycle 40 clone 1	[7–9]	E2	-	-	-
MyxoEE-3 P04 cycle 40 clone 1	[7–9]	E3	-	rifampicin	-
MyxoEE-3 P10 cycle 40 clone 1	[7–9]	E4	-	rifampicin	-
MyxoEE-3 P12 cycle 40 clone 1	[7–9]	E5	-	rifampicin	-
MyxoEE-3 P32 cycle 40 clone 1	[7–9]	E6	-	rifampicin	-
MyxoEE-3 P36 cycle 40 clone 1	[7–9]	E7	-	rifampicin	-
MyxoEE-3 P38 cycle 40 clone 2	[7–9]	E8	-	rifampicin	-
MyxoEE-3 P40 cycle 40 clone 1	[7–9]	E9	-	rifampicin	-
Chihaya 01	[10]	N1	-	-	Japan
Chihaya 20	[10]	N2	-	-	Japan
Colombia 01	[10]	N3	-	-	Colombia
Colombia 03	[10]	N4	-	-	Colombia
Nei 05	[10, 11]	N5	-	-	Mongolia
Nei 10	[10, 11]	N6	-	-	Mongolia
New Jersey 06	[10, 11]	N7	-	-	New Jersey, USA
New Jersey 10	[10, 11]	N8	-	-	New Jersey, USA
Serengeti 01	[10]	N9	-	-	Tanzania
Serengeti 21	[10]	N10	-	-	Tanzania
Sulawesi 08	[10, 11]	N11	-	-	Indonesia
Tubingen C22	[10, 11]	N12	-	-	Germany
Tubingen C42	[10, 11]	N13	-	-	Germany
GH2.1.4c40	[12]	N14	-	-	Indiana, USA (“Greg’s House”)
GH3.2.7C	[12]	N15	-	-	Indiana, USA (“Greg’s House”)
GH3.5.6c2	[12]	N16	-	-	Indiana, USA (“Greg’s House”)

GH5.1.9c20	[12]	N17	-	-	Indiana, USA (“Greg’s House”)
KF2.1.1B	[12]	N18	-	-	Indiana, USA (“Kent Farms”)
KF3.2.8c11	[12]	N19	-	-	Indiana, USA (“Kent Farms”)
KF5.4.6c29	[12]	N20	-	-	Indiana, USA (“Kent Farms”)
MC3.1.9c3	[12]	N21	-	-	Indiana, USA (“Moore’s Creek”)
MC3.2.6B	[12]	N22	-	-	Indiana, USA (“Moore’s Creek”)
MC3.5.9c15	[12]	N23	-	-	Indiana, USA (“Moore’s Creek”)
GJV1_*csgA413*	this work	GJV1-*csgA*	*csgA*::pCR-*csgA413*	kanamycin	-
Chihaya 20_*csgA413*	this work	N2-*csgA*	*csgA*::pCR-*csgA413*	kanamycin	-
Serengeti 01_*csgA413*	this work	N9-*csgA*	*csgA*::pCR-*csgA413*	kanamycin	-
GH3.5.6c2_*csgA413*	this work	N16-*csgA*	*csgA*::pCR-*csgA413*	kanamycin	-
MC3.5.9c15_*csgA413*	this work	N23-*csgA*	*csgA*::pCR-*csgA413*	kanamycin	-
GJV1_*csgA200*	this work	*csgA*-200	*csgA*::pCR-*csgA200*	kanamycin	-
GJV1_*csgA300*	this work	*csgA*-300	*csgA*::pCR-*csgA300*	kanamycin	-
GJV1_*csgA400*	this work	*csgA*-400	*csgA*::pCR-*csgA400*	kanamycin	-

[a]Strain DK1622 – the lab progenitor of both DK5208 and GJV1 – is reported to be derived from a strain isolated in Ames, Iowa, USA [13].
[b]Multiple clones were selected and stored frozen after MyxoEE-3 cycle 40.

Table S2. History and mutations of MyxoEE-3 lab-evolved strains. The strains referred to here as E1-E9 are clones from populations of *M. xanthus* that evolved as vegetatively growing colonies swarming across hard (1.5%) or soft (0.5%) agar [7, 8] over 40 two-week growth cycles. Here we provide information about the evolution conditions and accumulated mutations of each strain (from Table S4 in [7]).

Strain	Original name	Agar concentration	Mutations	Genic	Coding	Synonymous	Intergenic
E1	P02 clone 1	1.5%	13	10	8	2	3
E2	P03 clone 1	1.5%	10	10	10	0	0
E3	P04 clone 1	1.5%	10	9	9	0	1
E4	P10 clone 1	1.5%	10	10	7	3	0
E5	P12 clone 1	1.5%	19	15	14	1	4
E6	P32 clone 1	0.5%	12	12	11	1	0
E7	P36 clone 1	0.5%	14	14	13	1	0
E8	P38 clone 2	0.5%	10	10	9	1	0
E9	P40 clone 1	0.5%	13	12	11	1	1

Table S3. Plasmids constructed in this work.

Plasmid name	*csgA* allele
pGJV1*csgA483*	GJV1
pChihaya20*csgA483*	Chihaya 20
pSerengeti01*csgA483*	Serengeti 01
pGH356c2*csgA483*	GH3.5.6c2
pMC359c15*csgA483*	MC3.5.9c15
pGJV1*csgA200*	GJV1
pGJV1*csgA300*	GJV1
pGJV1*csgA400*	GJV1

SI References

1. Shimkets LJ, Asher SJ. Use of recombination techniques to examine the structure of the *csg* locus of *Myxococcus xanthus*. Mol Gen Genet. 1988;211:63–71.
2. Manhes P, Velicer GJ. Experimental evolution of selfish policing in social bacteria. Proc Natl Acad Sci. 2011;108:8357–62.
3. Velicer GJ, Kroos L, Lenski RE. Loss of social behaviors by *Myxococcus xanthus* during evolution in an unstructured habitat. Proc Natl Acad Sci. 1998;95:12376–80.
4. Velicer GJ, Raddatz G, Keller H, Deiss S, Lanz C, Dinkelacker I, et al. Comprehensive mutation identification in an evolved bacterial cooperator and its cheating ancestor. Proc Natl Acad Sci. 2006;103:8107–12.
5. Fiegna F, Yu YN, Kadam S V, Velicer GJ. Evolution of an obligate social cheater to a superior cooperator. Nature. 2006;441:310–4.
6. Zee PC, Mendes-Soares H, Yu Y-TN, Kraemer SA, Keller H, Ossowski S, et al. A shift from magnitude to sign epistasis during adaptive evolution of a bacterial social trait. Evolution. 2014;68:2701–8.
7. Rendueles O, Zee PC, Dinkelacker I, Amherd M, Wielgoss S, Velicer GJ. Rapid and widespread de novo evolution of kin discrimination. Proc Natl Acad Sci. 2015;112:9076–81.
8. Rendueles O, Velicer GJ. Evolution by flight and fight: diverse mechanisms of adaptation by actively motile microbes. ISME J. 2017;11:555–68.
9. Rendueles O, Velicer GJ. Hidden paths to endless forms most wonderful: Complexity of bacterial motility shapes diversification of latent phenotypes. BMC Evol Biol. 2020;20:145.
10. Rajagopalan R, Wielgoss S, Lippert G, Velicer GJ, Kroos L. *devI* Is an Evolutionarily Young Negative Regulator of *Myxococcus xanthus* Development. J Bacteriol. 2015;197:1249–62.
11. Vos M, Velicer GJ. Isolation by Distance in the Spore-Forming Soil Bacterium *Myxococcus xanthus*. Curr Biol. 2008;18:386–91.
12. Kraemer SA, Velicer GJ. Endemic social diversity within natural kin groups of a cooperative bacterium. Proc Natl Acad Sci. 2011;108:10823–30.
13. Dey A, Vassallo CN, Conklin AC, Pathak DT, Troselj V, Wall D. Sibling Rivalry in *Myxococcus xanthus* Is Mediated by Kin Recognition and a Polyploid Prophage. J Bacteriol. 2016;198:994–1004.
